# Application of Artificial Intelligence in Diagnosis of Craniopharyngioma

**DOI:** 10.3389/fneur.2021.752119

**Published:** 2022-01-06

**Authors:** Caijie Qin, Wenxing Hu, Xinsheng Wang, Xibo Ma

**Affiliations:** ^1^Institute of Information Engineering, Sanming University, Sanming, China; ^2^University of New South Wales, Sydney, NSW, Australia; ^3^School of Information Science and Engineering, Harbin Institute of Technology at Weihai, Weihai, China; ^4^CBSR & NLPR, Institute of Automation, Chinese Academy of Sciences, Beijing, China; ^5^School of Artificial Intelligence, University of Chinese Academy of Sciences, Beijing, China

**Keywords:** craniopharyngioma, tumor, diagnosis, machine learning, deep learning

## Abstract

Craniopharyngioma is a congenital brain tumor with clinical characteristics of hypothalamic-pituitary dysfunction, increased intracranial pressure, and visual field disorder, among other injuries. Its clinical diagnosis mainly depends on radiological examinations (such as Computed Tomography, Magnetic Resonance Imaging). However, assessing numerous radiological images manually is a challenging task, and the experience of doctors has a great influence on the diagnosis result. The development of artificial intelligence has brought about a great transformation in the clinical diagnosis of craniopharyngioma. This study reviewed the application of artificial intelligence technology in the clinical diagnosis of craniopharyngioma from the aspects of differential classification, prediction of tissue invasion and gene mutation, prognosis prediction, and so on. Based on the reviews, the technical route of intelligent diagnosis based on the traditional machine learning model and deep learning model were further proposed. Additionally, in terms of the limitations and possibilities of the development of artificial intelligence in craniopharyngioma diagnosis, this study discussed the attentions required in future research, including few-shot learning, imbalanced data set, semi-supervised models, and multi-omics fusion.

## 1. Introduction

### 1.1. Introduction of Craniopharyngioma

Craniopharyngioma is a common skull congenital tumor in clinical which accounts for 1.2–4.0% of all primary skull tumors (1). Its annual incidence rate is reported about 0.05–0.2 per 100,000 individuals (2). Craniopharyngioma has a wide range of age at onset even in the prenatal and neonatal period (3, 4). Craniopharyngioma occurs in a bimodal age distribution, with peak onset ages ranging from 5 to 14 years and 50 to 74 years (5).

The embryonic remnant theory is generally accepted for the pathogenesis of craniopharyngiomas. This theory believes that craniopharyngioma arises from the embryonic enamel primordium, which is located between the Rathke capsule and the oral craniopharyngeal tube, and is formed by residual epithelial cells remaining from craniopharyngeal duct insufficiency (6, 7).

The clinical manifestations of craniopharyngioma are diverse, depending on the tumor location, size, growth pattern, and the relationship with adjacent brain tissue. Craniopharyngioma grows slowly along the suprasellar, sphenoid sinus, posterior nasopharyngeal wall to the third ventricle, thereby forming compression on adjacent brain tissue and causing clinical manifestations including: (1) Symptoms of increased intracranial pressure, such as headache, vomiting, etc. (8). (2) Sudden changes in visual field and vision (9), which are caused by compression of the optic chiasmatic nerve because of suprasellar lesion. (3) Growth and developmental disorders, and decreased basal metabolic rate (10, 11), which are caused by insufficient secretion of growth hormone and gonadotropins because of the compression of the anterior pituitary gland. (4) As the tumor grows up to the suprasellar even to the bottom of the third ventricle, the hypothalamus is compressed and damaged. As a result, lethargy or even coma (12), electrolyte disturbance (13), diabetes insipidus (14), obesity (15), alterations of BcT ° (body core temperature) and sleep wake cycle rhythms (16), and other atypical symptoms may occur.

### 1.2. Radiomics

Although it is defined as a benign tumor by the World Health Organization (WHO), craniopharyngioma may cause significant morbidity and mortality due to its locally aggressive growth pattern (17). Therefore, early and accurate diagnosis of craniopharyngioma has important significance for the formulation of therapeutic schemes. Surgical pathological diagnosis is the current golden standard for diagnosing craniopharyngiomas, but it is lowly accepted by patients due to its invasive, high expense and complex operation. Besides, it is not easy to detect brain tissue invasion by histopathology because of the lack of brain tissue samples (18). The inaccurate diagnosis may affect the patient's therapeutic schemes and prognosis, and thus histopathology is difficult to apply to routine clinical examination. Instead of focusing on local tiny tissues, medical imaging can provide a more comprehensive view of the tumor. At present, medical imaging examinations mainly rely on neuroradiologists' subjective judgement on tumor tissues, they are time-comsuming, inefficient, and have subjective bias. With the development of artificial intelligence, radiomics can extract a large amount of image information through imaging methods, such as computed tomography (CT), magnetic resonance imaging (MRI), positron emission tomography (PET), and transformation of visual image information into deep-level features, which can quantitatively describe the image (19, 20). The deeper mining and analysis of numerous image information can assist neuroradiologists to make accurate diagnoses. The combination of radiomics and artificial intelligence methods have the advantages of being non-invasive, economical, efficient, and reproducible, thus can be widely used in tumor diagnosis, treatment, monitoring, and individualization of treatment.

### 1.3. Artificial Intelligence

Artificial intelligence (AI) is a multidisciplinary and interdisciplinary research on the basis of computer science, which applies the theory and method of simulating and expanding human intelligence to every field of life (21). The application of AI in the field of medical imaging can shorten the image processing time and improve the reliability of diagnostic results leveraging big data (22, 23). AI falls into the categories of traditional machine learning (ML) and deep learning (DL). The ML method inputs training data into the computer, gradually learns rules and recognition patterns based on big data, and finally analyzes the characteristic indicators to predict on new data. ML is characterized by the need to manually design a feature extractor to transform the original data into appropriate feature vectors, which has great influence for the prediction of new data (24). As an important branch of machine learning, deep learning has shown excellent performance in the field of image recognition (25). DL is a multi-level neural network model that combines low-level features to form high-level features, and then discovers the inherent characteristics of the data. It relies on the deep neural network to simulate human brain learning and analyzing data. Meanwhile, DL is also an algorithm highly dependent on big data, whose performance is enhanced with the increase in the amount of data and training intensity.

Although some attempts in the field of intelligent diagnosis of craniopharyngioma have emerged in recent years, the research of artificial intelligence in the diagnosis of craniopharyngioma is still in the preliminary stage. To this end, this study reviewed the existing research on intelligent diagnosis methods for craniopharyngioma, and introduced these applications of artificial intelligence technology in the diagnosis of craniopharyngioma from the aspects of differential classification, tissue invasiveness, gene mutation, and postoperative prediction. With reference to literature of AI in craniopharyngiomas and other similar tumors, this study proposed the technical route for intelligent diagnosis of craniopharyngiomas, focusing on MRI-based machine learning and deep learning methods. In the future research, it requires attentions, but not limited to, few-shot learning, imbalanced data set, semi-supervised learning, and multi-omics research.

The rest of this review was structured as follows: section 2 reviewed the applications of artificial intelligence in the diagnosis of craniopharyngioma from three aspects: differential classification, tissue invasion and gene mutation prediction, and prognosis prediction. Section 3 discussed in depth the intelligent diagnosis route of craniopharyngioma, including traditional machine learning and deep learning models, and a mixture of the two. Section 4 expounded the factors that affect the development of artificial intelligence technology in this field, and the attentions required for future research. Finally, the conclusion of the article was given in the last section.

## 2. The Applications of AI in Craniopharyngioma Diagnosis

Owing to the diversity of tumor shapes and types, craniopharyngiomas have different pathogeneses, degrees of malignancy, and therapeutic schema. Manual diagnosis is time-consuming in clinical practice, and may produce inconsistent results due to individual differences in patients and doctors' experience. Some research into the diagnosis of craniopharyngiomas based on artificial intelligence has emerged in recent years. In this retrospective study, Web of Science, Google Scholar and PubMed electronic databases were searched up to July 15, 2021. Other possible articles were searched manually from the citation list provided with each article. The potential literature searches were performed using the following keywords: “craniopharyngioma” AND “Artificial intelligence,” “craniopharyngioma” AND “machine learning,” “craniopharyngioma” AND “deep learning,” “craniopharyngioma” AND “non-invasive,” “craniopharyngioma” AND “MRI,” “craniopharyngioma” AND “diagnosis.”

### 2.1. Differential Classification

(1) Tian et al. (26) employed statistical methods to investigate the role of qualitative features and texture features on MRI between craniopharyngioma and meningioma. The study cohort was a single institutional database consisting of 127 patients with craniopharyngioma or meningioma. Doctors from relevant departments collaborated to evaluate MRI features qualitatively, which include signal intensity, heterogeneity, cystic changes, unenhanced area, the presence of air-fluid level, and the size and location of the tumor. Besides, LifeX medical software was used to extract texture features, including histogram-based matrix (HISTO), gray-level co-occurrence matrix (GLCM), gray-level run length matrix (GLRLM), etc. In this study, according to the previous reports, 10 of the most commonly used texture features were selected for analysis. IBM SPASS software and MedCalc were utilized for statistical analysis. Chi-square tests, Fisher exact test, and the Mann-Whitney *U*-test were used to evaluate the differences between two types of tumors. Additionally, binary logistic regression was adopted to predict the probability of texture feature as an independent predictor. The statistical results demonstrated that there were significant differences in five features between the two types of tumor, including HISTO-Skewness, GLCM-Contrast, GLCM-Dissimilarity on contrast-enhanced images, HISTO-Skewness, and GLCM-Contrast of T2-weighted imaging (T2WI). Later, in the logistic regression experiment, it was found that HISTO-Skewness, GLCM-Contrast on contrast-enhanced images, and HISTO-Skewness of T2WI can be used as independent predictors. The statistical methods facilitate better understanding of the data used for training, and enhance the interpretability of the machine learning model.

(2) Zhang et al. (27) reviewed the data of 126 patients with craniopharyngioma or pituitary adenoma from a single institution. Qualitative MRI features mentioned in previous reports were analyzed. Meanwhile, LifeX software was used to extract 46 texture features of the tumor, including HISTO, GLCM, GLRLM, GLZLM (gray-level zone length matrix), and NGLDM (Neighborhood gray-level dependence matrix). MRI features were evaluated by using chi-square test or Fisher test, while texture features were evaluated by using Man-Whitney test. Subsequently, binary logistic regression analysis was used to evaluate whether the significant features could be used as independent predictors. All statistical analyses were performed with SPSS software. The analysis results showed that the qualitative and textural features of MRI were of potential value in the differential diagnosis of craniopharyngioma and pituitary adenoma, which was helpful for clinicians to make decisions. The main limitation of the study appeared as a small database in a single institution with the exception of inevitable selection bias. On the other hand, the results may be affected by the different image characteristics between two types of craniopharyngioma. Besides that, the study did not evaluate the correlation between texture characteristics and pathology of tumor.

(3) Zhang et al. (28) adopted a machine learning model to identify common lesions presented in the anterior skull base with radiological parameters and clinical parameters. A single-institution database of 235 patients with pathologically proven pituitary adenoma, craniopharyngioma, meningioma, or Rathke fissure cyst were involved in the study cohort. Doctors from relevant departments utilized LifeX software to extract 40 texture features from an MRI, combined with clinical parameters (age, gender, etc.) to identify tumor types. In order to screen out more relevant feature sets, five feature selection methods were adopted, including distance correlation, random forest (RF), least absolute shrinkage, and selection operator (Lasso), extreme gradient boosting and gradient boosting decision tree (GBDT). In addition, nine classification models were employed for classification, including linear discriminant analysis (LDA), support vector machine (SVM), RF, Adaboost, k-nearest neighbor (KNN), Gaussian Naive Bayes (GaussianNB), logistic regression (LR), GBDT, and decision tree (DT). To evaluate the performance of machine learning models, indicators such as ROC, accuracy, sensitivity, and specificity were adopted. The SPSS software was utilized for statistical analysis, and the Python platform and the Scikit-Learn package were utilized to simulate machine learning algorithms. Among the 45 diagnostic models, the combination of LASSO and LDA achieved the best comprehensive effect, which had been reported in previous studies with good classification performance. However, the study cohort, simply from a single institution, was relatively small. In addition, radiomics analysis only adopted contrast-enhanced T1WI without other subsequences. Multi-model imaging statistics should be integrated into the study in future research.

(4) Prince et al. (29) tried adopting deep learning to identify craniopharyngiomas. The imaging data were obtained via Children's Hospital in Colorado. Experiments were conducted by using CT scan images and contrast-enhanced T1-weighted MRI. In order to overcome the disadvantages of small data sets, the study adopted a transfer learning method to obtain the pre-training model of ImageNet through the TensorFlow application module. Another measure to solve over-fitting problems was a three-term loss function comprised of sigmoid focal cross-entropy, triplet hard-loss, and CORellation alignment, also the effectiveness of the modified loss function was verifed in this study. In addition, a meta-heuristic parameter optimization method was adopted to mitigate the calculation loss of the model. The StandardScale function of Scikit-Learn was used to preprocess the image, and the Long Short-Term Memory model (LSTM) was employed for classification of adamantinomatous craniopharyngioma with other sellar/suprasellar tumors. The deep learning algorithm was based on the TensorFlow framework, which was written and executed by Python 3.6. The experimental results provided a transferable and extensible computing framework for intelligent diagnosis of rare diseases. Further optimization of classifiers will be the next potential. Also, training on MRI and CI simultaneously may acquire a more robust classification model. Furthermore, the integration of classification model into a lightweight web-based application will accelerate deployment to the clinical medical community.

(5) Prince et al. (30) studied a series of optimization methods to address small data sets and subsequently adopted deep learning algorithm to identify the pediatric adamantinomatous craniopharyngioma. Transfer learning was an effective technique to deal with overfitting of small data sets, which was verified in the study. In addition, two data augmentation techniques were utilized to expand the data set. One was the random data augmentation technique, which used random probability thresholds to transform the image, through cutting, rotation, blurring, etc. The other method was transformation adversarial networks for data augmentation (TANDA), which was an unsupervised image generation mode based on generative adversarial networks (GAN). Moreover, a meta-heuristic parameter optimization was applied to reduce the computational time. The experimental cohort involved a small data set from Children's Hospital Colorado and St. Jude Children's Research Hospital. The program was developed in the virtual environment of python 3.6. The results showed that the performance of the optimal model was comparable to the average of radiologists, and the deep learning network achieved the best performance with the combination of CT and MRI data sets. Potential next steps include optimization of TANDA algorithm and synthetically expanding the data set which could leverage pre-trained feature extraction. Besides that, the study adopted only one type of classification model. Other additional classifiers should be explored in future research.

### 2.2. Prediction of Tissue Invasion and Gene Mutation

(1) Although Craniopharyngioma is a benign tumor, it is still possible to invade adjacent brain tissue which results in incomplete surgical resection and poor prognosis. Therefore, preoperative evaluation of the tissue invasion of craniopharyngioma is helpful to formulate a more individualized surgical scheme. Ma et al. (31) used a machine learning algorithm combined with radiological characteristics to predict preoperative craniopharyngioma invasiveness. The study cohort consisted of 325 patients in a single institution. The researchers utilized MRIcron software to manually delineating the region of interest, and applied Z-score transformation on the images to avoid heterogeneity bias. A total of 1,874 features were extracted in this study. After being screened by using the Lasso feature selection method, an optimal feature subset of 11 features was finally selected and fed into the SVM model for invasion prediction. The experiments used the ROC curve to evaluate the performance of the learning model. The results suggested that this non-invasive radiomics approach could predict the invasiveness of craniopharyngioma, aid clinical decision making, and improve patient prognosis.

(2) Craniopharyngiomas are classified into two histologic subtypes: Adamantinomatous craniopharyngioma (ACP) and papillary craniopharyngioma (PCP). BRAF and CTNNB1 mutations are found to be strongly correlated with the pathological subtypes of craniopharyngiomas, which means the diagnosis of pathological subtypes and gene mutations has great significance for the effective adjuvant targeted therapy. Chen et al. (32) discussed the prediction of BRAF and CTNNB1 mutations through radiomics method based on MRI. The study reviewed the preoperative MRI data of 44 patients with craniopharyngioma from a single study institution. In this study, 464 local features were obtained using quantitative location evaluation methods, and another 555 high-throughput features including intensity, shape, texture, and wavelet features were extracted by using MATLAB tools. In order to reduce the redundancy and computational complexity of features, a three-stage feature selection method was adopted. In the first stage, High-throughput texture features were evaluated according to intra-class correlation coefficients (ICC), and the features with ICC greater than 0.8 remained. In the second stage, the data set was sampled 100 times by the bootstrap, and the feature selection method embedded in Random Forest model was adopted to preserve the highest average of results. In the third stage, sequence forward selection strategy was applied to evaluate the prediction effect of candidate feature subsets according to the performance of Random Forest classification. Finally, the most relevant feature subset was screened out and fed into the Random Forest model, and then the performance of the classification model was evaluated by 10-fold cross-validation. The method proposed in this study achieved considerable accuracy in the prediction of pathological subtypes and the classification of gene mutation status.

(3) The cystic part of craniopharyngioma can easily aggress adjacent brain tissue, making complete surgical removal difficult. Since CTNNB1 mutation has been proved to be related to tumor recurrence, the prediction of CTNNB1 mutation in craniopharyngioma can facilitate surgical treatment and reduce postoperative recurrence rates with molecular targeted drugs. Zhu et al. (33) adopted the preoperative MRI data of children with cystic ACP from a institutional database, quantitatively measured the MRI by the picture archiving and communication system (PACS), and extracted the location, quantity, shape, maximum diameter, internal signal, cyst wall, and other characteristics. Continuous data were assessed through Mann-Whitney *U*-test, categorical data were analyzed through Fisher's exact test, and all the statistical analyses were carried out by using SPASS software. The study confirmed the differences in MRI features between patients with CTNNB1 mutation and the control group. Through the identification of gene mutations, appropriate preoperative inhibitors may prevent the formation of cystic tumors and reduce the size of tumors, thereby providing the best opportunity for surgical treatment.

(4) Prediction of BRAF mutation before surgery and treatment with inhibitors may shrink the tumor and improve the success rate of surgical resection. Yue et al. (34) dedicated their research to the study of the non-invasive diagnosis method of BRAF mutation in craniopharyngioma. The study reviewed the information of patients with craniopharyngioma from a single institution, and MRIs (including non-enhanced sequences and contrast-enhanced sequences) of 52 patients were involved in the study. The study assessed MRI features including tumor location, size, shape, composition, tumor cysts signal, enhancement pattern, pituitary stalk morphology, and internal carotid artery encasement. A Mann-Whitney test was adopted to evaluate the continuous variables, Fisher's exact test was used to compare the categorical variables, and these statistical analyses were carried out by using SPSS software. The results showed that five features of the MRI were related to BRAF mutation, and this non-invasive diagnostic approach provided a reference for the use of targeted inhibitors before surgery.

(5) Due to the heterogeneity in clinical expression, topography, and pathological features of craniopharyngioma, the diagnosis and surgical treatment of craniopharyngioma are still challenging in the clinic. Craniopharyngioma (CP) may occur at any point from the sella to the third ventricle, along the vertical hypothalamic-pituitary axis. The anatomical relationship between craniopharyngioma and the third ventricle is a vital factor for surgical schemes. Prieto et al. (35) discussed the topographic classification of craniopharyngioma with preoperative MRI. In the study, the tumors were classified into five major categories, including sellar-suprasellar CPs, suprasellar-pseudoventricle CPs, secondary intraventricular CPs, infundibulo-tuberal CPs, and strictly intraventricular CPs. The study retrospected the MRI of 200 craniopharyngiomas selected from a recent publication, and analyzed radiologic features related to tumor size, shape, consistency, the occupation of the tumor of intracranial compartments, the distortions of anatomic structures along the sella turcica–third ventricle axis, etc. The correlations between pairs of categorical variables were evaluated by using chi-square or Monte Carlo validation, then the topographic classification of craniopharyngioma was explained through multiple classification and regression tree growing method. The statistical analysis was carried out by using SPSS software. The experiment result identified seven radiologic features on preoperative MRI which should be analyzed to accurately define the topography of CP. A further step may be the integration of specific MR imaging sequences which can offer high-resolution.

### 2.3. Prognosis

Due to the tissue invasion, surgical resection of craniopharyngioma is often incomplete, which leads to tumor recurrence and poor prognosis. The prediction models of prognosis require complex and abundant data, hence artificial intelligence technology is appropriate to traditional statistical methods. In this field, some research on the prognosis of brain tumors such as pituitary tumors have been emerging. Inspired by these attempts, some researchers studied the postoperative prediction of craniopharyngioma. Hollon et al. (36) predicted early outcomes of pituitary adenoma surgery with a machine learning model. In the study, a retrospective review was constructed for a cohort of 400 consecutive pituitary adenoma treated at a tertiary care center. Naive Bayes, logistic regression with elastic net (LR-EN) regularization (linearly combined L1 and L2 regularization penalties), SVM, and random forest were adopted to predict early postoperative outcomes in pituitary adenoma patients. The experiment selected twenty-six characteristics as predictive variables, used a grid search for the selection of model hyperparameters, and performed a 10-fold cross-validation for each model. After model training, LR-EN best predicted early postoperative outcomes with 87% accuracy. Additionally, risk factors for postoperative complications after pituitary adenoma surgery were explored. Moreover, these results provided insight into how to use machine learning models to improve the perioperative management of pituitary adenoma patients.

Shahrestani et al. (37) developed a multilayered neural network (NN) to estimate predictors of postoperative complications and outcomes in patients with functional pituitary adenomas (FPAs). Three hundred forty-eight patients with FPAs in a single center were included in the analysis. First, the study performed multivariate regression model to test the correlation between patient-specific characteristics and good outcomes. Then, the NNs with strong ability in non-linear models were trained on significant variables obtained in multivariate analysis. Weights and bias terms were calculated from these back-propagation models. The NN models were tested and confirmed by using ROC curve, AUC value, and confusion matrices. The study developed a robust prediction algorithm for recurrence, progression, and hormonal non-remission in patients with FPAs.

Since craniopharyngioma is structurally close to the optic nerve, visual dysfunction occurs in 53–93% of patients. Kopparapu et al. (9) collected preoperative, intraoperative, and postoperative variables of craniopharyngioma in a single institution, including demographics, radiology information, surgical approach, etc. Meanwhile, radiographic, operative, and perioperative characteristics were qualified in this study. Besides, the patient's visual characteristics such as visual acuity (VA) and visual fields (VFS) were standardized according to the guidelines defined by the German Ophthalmological Society. Statistical analyses were performed by using Stata software. Categorical variables were analyzed through chi-square test, and continuous variables were analyzed through independent-sample *t*-test. Furthermore, multivariate analysis was carried out by using logistic regression for significant variables. The analysis result demonstrated that patients with reduced preoperative visual acuity, specific readiographic vascular involvement, and total surgical resection had more possibility of achieving improved postoperative visual acuity. In addition, statistical analysis also found that the translaminar surgical approach was related to visual deterioration. Postoperative vision prediction is helpful for counseling between patients and surgeons and may facilitate the customization of surgical schemes. The limitations of the study are consistent with other similar retrospective reviews. A relatively small sample size affected the results of study and resulted in non-significant findings on bivariate and multivariate analysis.

## 3. Strategies of Artificial Intelligence in Craniopharyngioma Diagnosis

In the field of clinical medical imaging, subjective diagnosis of radiologists has inevitable bias and is time-consuming. Moreover, it is a challenge for medical staff to address numerous data and high-dimensional features manually. With the development of artificial intelligence technology and its successful applications in many fields, more and more research has been performed on the application of artificial intelligence in the medical field. As shown in section 2 above, some attempts emerged in differential classification, prediction of tissue invasiveness and gene mutation, and prognosis detection of craniopharyngioma. However, these works represent an initial exploration in the diagnosis of craniopharyngioma. Glioma and lung adenocarcinoma, similar to craniopharyngioma in tissue invasion, with which difficulties in diagnosis and individualization of treatment are also associated. Glioma is one of the most aggressive brain tumors with poor prognosis compared with other brain cancers. Diffuse invasion of tumor cells into normal brain is a big challenge in diagnosis and individualization of treatment (38). Lung cancer is a common cause of cancer incidents worldwide, comprising 1/3 to 1/2 of incidents being attributed to adenocarcinoma. Due to an increased degree of invasion with poor prognosis, evaluation of invasion of lung nodules is important to customize the appropriate clinical-decision scheme (39). Thus, many studies have been dedicated to developing a new, non-invasive method that provided a reference for accurately diagnosis before surgery to reduce tumor recurrence and improve patients prognosis. Inspired by this research, the study referred to the achievements of AI diagnostic techniques for tumors (e.g., glioma, lung adenocarcinoma), and furthermore proposed the diagnosis route of craniopharyngioma based on artificial intelligence technology.

At present, imaging examinations are common methods for clinical diagnosis of craniopharyngioma. Compared with CT, MRI can clearly display the tumor location and the anatomical relationship of adjacent tissues through multi-directional imaging. The aforementioned studies also found that the features extracted from MRI have significant difference for the diagnosis of craniopharyngioma (26, 35). Therefore, the artificial intelligence diagnosis route discussed in this study is based on MRI.

### 3.1. Machine Learning Mode

**Figure 1:**
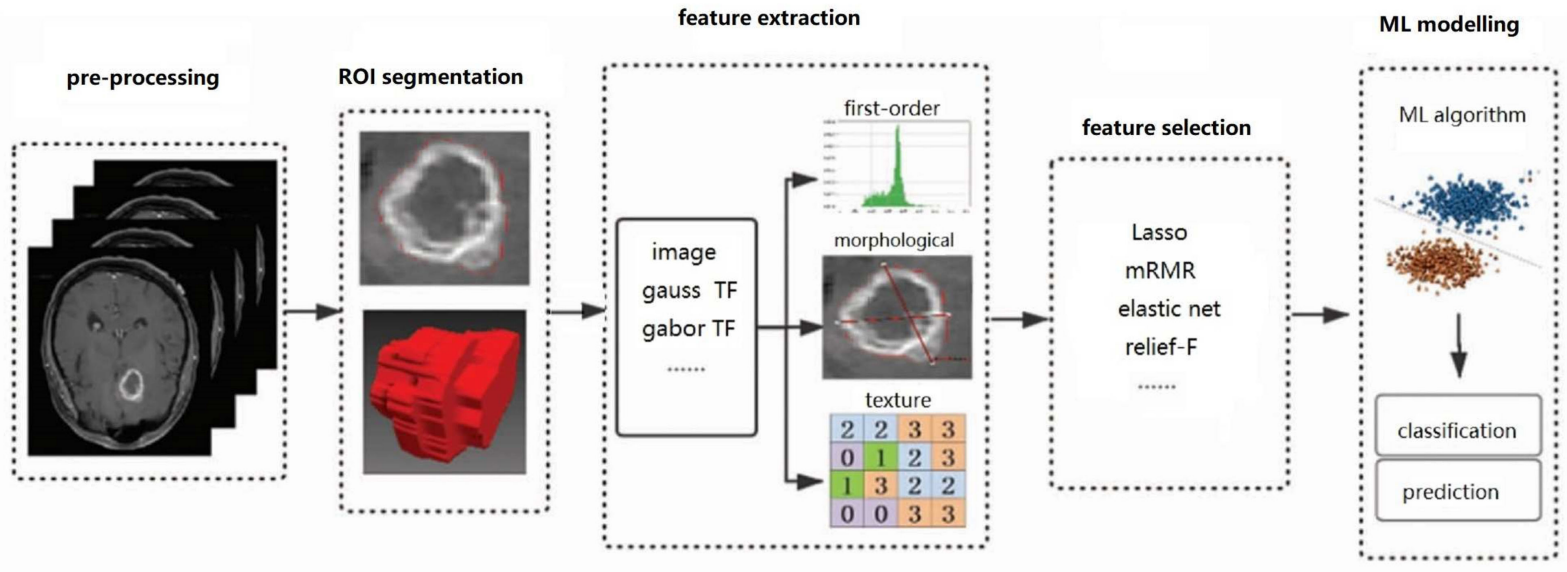
Flow chart of diagnostic craniopharyngioma based on ML: the steps include pre-processing, ROI segmentation, feature extraction, feature selection, and ML modeling. “TL” is the abbreviation of transformation.

While deep learning has become the technology of choice for most AI problems, it relies excessively on large data sets, which are difficult to collect, expensive, and time-consuming, especially for scarce tumors like craniopharyngioma. For small data sets, classical machine learning methods sometimes outperform deep learning models, with lower computational cost, and unnecessary high-end hardware. On the other hand, deep learning is a “black box.” In comparison, machine learning algorithms are more explanatory. The diagnostic route based on machine learning method is shown in Figure 1, which mainly includes image pre-processing, image region of interest (ROI) segmentation, feature extraction, feature selection, machine learning modeling, and other steps.

(1) Image pre-processing: As the acquisition of image data is dependent on device manufacturer, device parameters and patient position, pre-processing techniques can correct and normalize original images to weaken the interference information, and also improved the quality of core areas in the image. For brain MRI, the general pre-processing techniques usually include noise suppression, skull stripping, non-uniform correction, intensity normalization, and so on.

Noise suppression and non-uniformity correction can reduce the individual differences of the collected objects and the MRI brightness differences caused by the deviation of the instrument and the scanning process. For image sequences with various intensity ranges of the same image, such as T1WI and T2WI, intensity normalization is required for preprocessing. Skull stripping as an important part of brain image analysis can mitigate non-uniform distribution of intensity due to fat tissue. Skull stripping can be automatically implemented base on contour or histogram except for when using manual methods. Some of the pre-processing techniques in literature are as follows: Al-Saffar and Yildirim (40) performed skull stripping with a threshold-based method, and adopted median filtering technology to reduce image noise. Gutta et al. (41) used BrainSuite software for resampling all images and skull-stripping, and used the FMRIB Software Library (FSL) toolbox for co-registration. Analogously, Ahammed et al. (42) also performed skull stripping by using BrainSuite software, which adopted a brain surface extraction algorithm (BSE) to operate the skull stripping. Aiming to solve the non-standardization of MRI intensity, Chen et al. (43) used bias correction and Z-score normalization which were implemented in Statistical Parametric Mapping (SPM12) for pre-processing. Siakallis et al. (44) performed log-transformation, normalization, bias field correction and intensity matching on skull stripping images. Kandemirli et al. (45) conducted pre-processing operations including gray-level normalization and discretization. The commonly used techniques and tools in representative literature are listed in Table 1.

**Table 1:** An overview of techniques for pre-processing based on MRI.

**References**	**Pre-processing**	**Tool**
Al-Saffar and Yildirim (40)	Skull stripping based on threshold,	MATLAB
	Median filtering for noise reduction	
Gutta et al. (41)	Skull stripping	Brainsuite software
KV et al. (42)	Skull stripping	Brainsuite software
Chen et al. (43)	Bias correction, Z-score normalization	SPM12
Siakallis et al. (44)	Logarithmic transformation, normalization,	
	Offset field correction, strength matching	
Kandemirli et al. (45)	Grayscale normalization, Discretization	

(2) ROI segmentation: Image ROI segmentation has an important impact on the final result of medical classification. ROI segmentation can remove surrounding tissues of the lesion and irrelevant interference information in the background, and identify the lesion area by describing the density, shape and other characteristics of the ROI. In many research studies, neuroradiologists manually delineate the ROI which requires deliberations to demonstrate the effectiveness of the segmentation and reach a consensus on the differences. In addition, semi-automatic or automatic segmentation algorithms are also feasible. Threshold segmentation, as a commonly used method, takes the gray value of the pixel as the feature description, and uses the threshold to distinguish background information and segmentation targets (46). A common method based on region, the image starts from a certain point and merges the surrounding pixel points with the same attribute (including gray value, texture and other features) (47). There are also some segmentation algorithms based on specific theories, such as minimized graph cut algorithm based on energy (48), conditional random field method based on statistics (49), and clustering analysis method based on fuzzy sets (50), etc. In addition, the deep learning network can automatically obtain features from the training data and achieve good segmentation performance (51, 52).

Part of the research performed manual segmentation by using ITK-SNAP, MRIcron, LifeX, and other softwares. Tekawa et al. (53) performed semi-automatic image segmentation by using the Analysis of Functional Neuro Images software (NUMH Scientific and Statistical Computing Core; Bethesda, MD, USA). In the semi-automatic segmentation procedure, the neuroradiologist manually selected the intensity threshold to extract the high-intensity areas of T2-weighted FLAIR images. In addition, there were some auto-segmentation methods adopted in retrieved literature. Al-Saffar and Yildirim (40) adopted the LDI-means clustering algorithm for image automatic segmentation. Ahammed et al. (42) combined k-means and fuzzy-c-means clustering method for automatic segmentation. Gutta et al. (41) performed segmentation with a fully automated tumor segmentation tool, which came from the Brain Tumor Segmentation Challenge. The aforementioned automatic segmentation methods also achieved good performance.

Different ROI segmentation techniques in representative literature are summarized in Table 2. Manual segmentation methods are simple and easy to master, but there are inevitably subjective differences. Some automatic segmentation algorithms with better performance can optimize the clinical diagnosis process, but their applications need to be evaluated with strict clinical tests.

**Table 2:** An overview of techniques for ROI segmentation based on MRI.

**References**	**Manual/ semi-automatic/ automatic**	**Method/tool**
Özyurt et al. (54)	Manual	MRIcron software
Tian et al. (26), Zhang et al. (27)	Manual	LIfeX software
Siakallis et al. (44)	Manual	ITK-SNAP 3.8
Tatekawa et al. (53)	Semi-automatic	Analysis of Functional Neuro Images software
Al-Saffar and Yildirim (40)	Automatic	LDI-means clustering algorithm
KV et al. (42)	Automatic	Combined k-means and fuzzy c-means
Gutta et al. (41)	Automatic	A tool from competition

(3) Feature extraction: This is a process of integrating, analyzing and calculating numerous features of ROI with various algorithms, which has a significant influence on the whole process. A high-quality feature set can not only simplify the complexity of the images sample, but also better represent the structural information, visual characteristics, and biological background knowledge of images, and directly affect the prediction effect of models. The features extracted from MRI commonly include first-order features, morphological features, texture features, and wavelet features (55), etc. First-order features, also known as histogram features, are extracted from the gray histogram of images. First-order features can only be used to describe the gray value distribution of ROI, but cannot describe the spatial relationship, interaction, and correlation between adjacent voxels. Morphological features can be used to quantitatively describe the geometric characteristics of ROI, which are good indicators of the anatomical changes of the tumor. Texture features represent the spatial arrangement between the image voxel gray levels, and facilitate evaluation of the heterogeneity inside the tumor. If two types of tumors have similar intensity distributions but different spatial correlations, second-order or higher-order texture features may be preferable to first-order features in such a situation. Texture features can also be obtained by the image Laplace transform, wavelet transform, Gabor transform, etc. Since a single feature set with insufficient information may lead to the risk of under-fitting in the training model, most literature use mixed feature sets in the research.

Commonly used image features are briefly described in Table 3, and the single or mixed feature sets extracted in the representative literature are summarized in Table 4.

**Table 3:** An introduction of feature extracted from MRI.

**Feature type**	**Feature family**
First-order features	Mean, maximum, minimum, median, root mean square, energy, entropy, kurtosis, skewness, variance, standard deviation, uniformity, gray field, etc.
Morphological features	Density, 3D maximum diameter, spherical asymmetry, sphericity, surface area, ratio of surface to volume, volume, etc.
Texture features	Gray-level co-occurrence matrix, gray-level run matrix, gray-level area size matrix, gray-level correlation matrix, adjacent gray- level difference matrix, neighborhood gray-level dependence matrix and gray-level run length matrix, etc.
Common transformations	Laplace transform, wavelet transform, Gabor transform, etc.

**Table 4:** An overview of features extracted from MRI and feature selection methods.

**References**	**Original feature set**	**Dimension**	**Extraction tool**	**Selection method**
Zhang et al. (28)	First-order features, GLCM, GLZLM, NGLDM, GLRLM, gender, age	40+2	LifeX	Distance Correlation, RF, Lasso, XGBoost, and GBDT
Ma et al. (31)	First-order statistics, shape, GLCM, GLRLM, GLSZM, NGTDM, GLDM, Wavelet features	1,874	MATLAB	Lasso
Gutta et al. (41)	First-order feature, shape, GLCM, GLRLM, GLSZM, NGTDM, GLDM	1,284	PyRadiomics	Importance score from gradient boosting algorithm
Le et al. (56)	Intensity, image derivative, geodesic, texture, posterior probability maps	704	Cancer Imaging Phenomics Toolkit	F-score evaluation criterion, recursive feature elimination
Al-Saffar and Yildirim (40)	GLCM, intensity	40		MI evaluation criterion, SVD
Kandemirli et al. (45)	Intensity, shape, GLCM, GLRLM, GLSZM, GLDM	3,255	Pyradiomics	Intraclass correlation coefficient, XGBoost's inherent feature selection and additional feature selection method
Gao et al. (57)	First order features, shape, GLCM, GLRLM, GLSZM	1,421	PyRadiomics	Chi2 verification, Seaborn library, inherent feature selection of RF
Chen et al. (32)	Local feature, intensity, shape, texture and wavelet features	1,091	MATLAB	Intraclass correlation coefficients, feature scores of RF, forward search strategy

*gray-level co-occurrence matrix; GLZLM, gray-level zone length matrix; NGLDM, neighborhood gray-level dependence matrix; GLRLM, gray-level run length matrix; GLSZM, gray-level size zone matrix; NGTDM, neighboring gray tone difference matrix; GLDM, gray-level dependence matrix*.

(4) Feature selection: The feature dimension obtained in the previous feature extraction process counts from tens to thousands, which leads to inevitable problems of computational complexity and overfitting. The feature selection method should be recommended to further optimize the high-dimensional features, eliminate redundant features, screen out the most relevant feature subsets, and avoid over-fitting. Commonly used feature selection methods include the criterion-based sorting methods that sort the features according to the evaluation criterion and select the feature with a higher score than the threshold. The general evaluation criteria include Fisher score (58), Pearson correlation coefficient (59), mutual information (60), etc. In addition, some heuristic rules can also be applied in selecting subsets, such as forward/backward search strategy (61), Markov chain (62), etc. It can also combine the criterion-based sorting method with the search strategy to form a two-step feature selection method, such as the feature selection method with maximum relation and minimum redundancy (63), which obtained good result in reports. There are also some learning algorithms with embedded feature evaluation, among which the decision tree is a typical algorithm (64).

Zhang et al. (28) verified five feature selection methods including distance correlation, random forest, Lasso, extreme gradient boosting and gradient boosting decision tree, of which Lasso achieved the best performance. And Ma et al. (31) also adopted Lasso for feature selection. Gutta et al. (41) used the importance score in the gradient boosting algorithm to select the features. Hybrid feature selection methods were adopted in some literature. Le et al. (56) employed a two-level feature selection method. In this method, the salient radiological features were evaluated with F-score criterion and ranked in descending order. Sequentially, the features were added to the model one by one, meanwhile the recursive feature culling technique was adopted to select the best cut-off point. Al-Saffar and Yildirim (40) adopted mutual information (MI) to evaluate the features, and used singular value decomposition (SVD) method to reduce feature dimension. Kandemirli et al. (45) firstly used the intraclass correlation coefficient to eliminate the features with score lower than 0.75, and then adopted the intrinsic feature selection method of XGBoost or additional feature selection methods (such as Boruta, low variance filter, and multicollinearity analysis) for further selection. Gao et al. (57) obtained the top 15 features according to Chi-square evaluate criterion, and drew a heatmap by using Seaborn library to identify the highly correlated features. Finally, the optimal feature subset was selected in terms of the feature importance of Random Forest algorithm. Chen et al. (32) proposed a three-stage feature selection method. In the first stage, intraclass correlation coefficients were used to screen robust variables. Sequentially, the robust texture features, location features, and clinical features were evaluated according to the feature score of Random Forest, and the top-ranked features were retained. In the third stage, a sequential forward search strategy was adopted to select the optimal feature subset.

The feature set and feature selection methods used in representative literature are compared in Table 4. Although there are a lot of mature feature selection methods, there are still problems involved in designing effective methods for specific scenarios. In the retrieved literature, Lasso is a linear regression model with the constraint term of L1 norm added behind the cost function. It carries out variable screening and complexity adjustment through the control parameter lambda, and is widely used in the medical field. In addition, the combination of different types of feature selection methods and the design of appropriate evaluation criterion according to specific scenarios are also required for further research.

(5) Machine learning modeling: In this procedure, a high-quality classifier is generated through sample training, and the feature vectors obtained after feature extraction and selection are fed into the classifier, thereby outputting the diagnosis results. Machine learning algorithms are classified as supervised learning and unsupervised learning. Supervised learning models are commonly used in the current medical field, such as random forest (RF), artificial neural network (ANN), support vector machine (SVM), logistic regression (LR), and so on.

The machine learning models used in representative literature are compared in Table 5. Based on the principle of maximum interval separation, SVM can mitigate the structural risk by minimizing the model complexity and training error, and has became an important tool for data classification in the field of pattern recognition. SVM can solve problems of high dimension and overfitting, and is often recommended in small sample classification scenarios.

**Table 5:** An overview of ML model in literatures.

**References**	**ML model**	**The best one**
Le et al. (56)	KNN, NB, RF, SVM, XGBoost	XGBoost
Al-Saffar and Yildirim (40)	Multi-layer perceptron, RBF-SVM	
Kaplan et al. (65)	KNN, ANN, RF, A1DE, LDA	KNN
Kandemirli et al. (45)	XGBoost	
Tatekawa et al. (53)	SVM	
Gao et al. (57)	LR, SVM, RF	RF
Zhang et al. (66)	LR, SVM	LR
Siakallis et al. (44)	SVM	
Zhang et al. (28)	LDA, SVM, RF, Adaboost, KNN, GaussianNB, LR, GBDT, DT	Lasso+LDA
Chen et al. (32)	RF	
Ma et al. (31)	SVM	

### 3.2. Deep Learning Mode

A machine learning algorithm requires complex feature engineering, which needs to explore and analyze the data set, reduce dimension of the feature set, and finally select the optimal feature subset to feed to the machine learning model. In contrast, the deep learning method is a kind of end-to-end learning, which has a strong learning ability and is easy to be transplanted. In many fields, deep learning algorithms perform far better than machine learning algorithms based on large amounts of data. Herein, craniopharyngioma diagnosis based on deep learning includes data augmentation and design of models.

(1) Data augmentation: Data is the driving force for deep learning models. Feeding abundant data into the training process can significantly improve the performance of the deep learning model. Whereas, in the field of medical research, due to the difficulty of data collection and the high cost of data labeling, the training data are not so sufficient, which results in poor model generalization and insufficient credibility of deep learning models. Data augmentation technology generates new data by transforming existing data, which is an important measure to expand the number of samples and improve the generalization ability of deep learning models. Therefore, data augmentation is widely used in the training process of deep learning models in the medical field. Data augmentation techniques can be implemented by transforming a single image or mixing information of multiple images. The geometric transformation commonly used in medical images is a typical data deformation operation (67), which generates new samples through rotation, mirroring, translation, cropping, etc. In addition, SMOTE, mixup and other methods can mix information from multiple images and synthesize new samples (68, 69). With the further development of artificial intelligence, novel data augmentation methods such as adversarial learning, meta-learning, and reinforcement learning have emerged and achieved good performance (70–72).

Data deformation technology was commonly utilized in most of the literature to achieve data augmentation, which can quickly and directly expand the data set. In (73), flips, rotation, shift, zoom, ZCA whitening, shearing, and brightness operations were used to achieve data augmentation. In (74), methods such as vertical flip, horizontal flip, image rotation of 90 degrees, 270 degrees, and image transpose were adopted to expand the data set by 6 times. In (75), Augmenter library was utilized to implement a cascade of rotation, zooming, shearing, and flipping techniques with certain probability, and elastic transformation was combined for data augmentation. As a result the training data set was increased by 20 times. In (76), eight different data augment techniques, including flip vertical, flip horizontal, rotate at 180 degree, rotation at 90 degree, noise, shear, blurr, and crop & scale were compared. The results showed that rotation at 90 degree and 180 degree achieved the best performance. In (77), contrast conversion, brightness conversion, sharpening and flipping were used to expand the data, and the training data was increased by four times. In (78), elastic transformation was used for data expansion. Autoaugment tool was used in (79), and multiple geometric transformation strategies were used to expand the data set to 23 times. In (80), the inversion technique in geometric transformation was adopted, and the classification accuracy was improved from 82.46 to 96.4% through data augmentation. In (81), data augmentation was carried out by using random clockwise rotation, counterclockwise rotation, and vertical flipping techniques.

Some literature also utilized GAN technology for data synthesis. For example, Carver et al. (82) used GAN network to generate high-quality images. Price et al. (30) employed two data augment techniques, one was a random process that used probability thresholds for sample transformations, and the other was a transformation adversarial network for data augmentation (TANDA). The relative simplicity of the image led to the advantage of random augment over TANDA. TANDA method was more advantageous for complex data sets where the target and background were indistinguishable.

Image deformation technology, as a basic image augmentation method, has been widely used for preprocessing in the field of image processing, and most of the methods are integrated into the machine learning library for deep learning applications. However, the transformation rules are not universal, and some of them only perform well on specific data sets. Data augmentation based on GAN which can generate the virtual image sample close to reality provides a new solution for data augmentation. More important, virtual images generated from noise images can enrich the randomness and diversity of samples, thus greatly improving the performance of the model. Nevertheless, the GAN training process is extremely complex and requires a large amount of computation, which limits its application in large image data sets. The techniques for data augmentation are listed in Table 6.

**Table 6:** An overview of techniques for data augmentation.

**References**	**Techniques for data augmentation**
Ismael et al. (73)	Flips, rotations, shifting, zooming, ZCA whitening, shearing, brightness manipulation
Zhang et al. (74)	Flips, rotations, image transpose
Özcan et al. (75)	A chain of rotation, zooming, shearing, flippling, and elastic transforms
Safdar et al. (76)	Flips, rotations, noise, shear, blurr, crop and scale
Wu et al. (77)	Contrast & brightness conversion, sharpening, flippling
Diaz-Pernas et al. (78)	Elastic transformation
Zhuge et al. (79)	Geometric transformation
Mzoughi et al. (80)	Geometric transformation
Atici et al. (81)	Rotations, flippin
Carver et al. (82)	GAN
Prince et al. (30)	TANDA, random transformations

(2) Design of deep learning model : Deep neural networks are developed on the basis of the early artificial neural network. Through the deeper neural network structure, the expression ability and the performance of the whole network have been greatly improved. With the breakthrough of deep learning in the traditional image recognition field, many classical deep learning frameworks have emerged, especially since large-scale image data sets became open source. In deep learning models, convolutional neural network can be considered as one of the most classic network models (83), which is usually composed of convolutional layer, pooling layer, full connection layer, etc. The convolutional layer obtains the feature information of the image through convolution operations, and synthesizes the local features to global features. The pooling layer is used to reduce the dimension of features and improve computational efficiency of the network. The full-connection layer combines the pooled multiple groups of features into a group of signals, and performs classification and recognition tasks through the classifier. Many classic networks have evolved from the convolutional neural network. For example, the AlexNet network uses rectifying linear units (RELU) to introduce non-linearity, adopts dropout technology to selectively ignore some neurons to avoid overfitting, and stacks maximum pooling layers to improve the disadvantage of average pooling (84). As a result, AlexNet can learn more complex object and hierarchical architectures. In addition, the VGG network from the University of Oxford adopts continuous and multiple 3*3 convolution to simulate the effect of larger convolution kernel (85). This technology can extract more complex features, but the drawback is the increase of parameter number and the requirement of computing power. Therefore, various network variants are derived to address this drawback. Typically, the ResNet adds cross-layer connections to form residual elements, which solves the problems of network degradation and gradient explosion caused by the deepening of the network layer (86). Additionally, in terms of the problem that a convolutional neural network can not extract and retain the time series, the recurrent neural network (RNN) maintains the dependency relationship in data through the serial structure (87), which is suitable for time series data. There are also other variants of deep learning networks that have also achieved good performance in the field of medical imaging (88, 89). Deep learning models in the representative literature are summarized as follows:

Özcan et al. (75) adopted CNN network comprised of 7 convolution layers, a full connection layer, and a classifier of Softmax function. The proposed model obtained high performance and robustness in glioma grading. Chang et al. (90) used a CNN network to classify the gene mutation of glioma. Francisco et al. (78) adopted a CNN network to differential diagnosis of meningioma, glioma, and pituitary tumor, and achieved a high classification accuracy. Mehmet et al. (81) adopted a CNN network to automatically detect high-grade glioma and achieved good performance. Prince et al. (30) adopted a CNN network with parameter optimizer to realize the non-invasive diagnosis of adamantinomatous craniopharyngioma. Wu et al. (77) employed three CNN network models (AlexNet, ResNet, Inception-V3) to classify glioma and encephalitis. The results of the automatic classification were compared with the performance of the radiologists and acquired satisfying performance. Mzoughi et al. (80) graded glioma by using a 3DCNN network, which fused local and global context information through a small convolution kernel. Wang et al. (91) developed and validated a 3DCNN model for classification of different types of lung cancer. The performance of the proposed classification model was compared to radiologists and obtained a higher score. Ismael et al. (73) identified three types of brain tumors (meningiomas, gliomas, and pituitary tumors) on MRI with the Resnet50 framework that is a 50-layer variant of the residual network. Zhuge et al. (79) proposed two automatic methods for glioma grading, one of which was a 3DConvNet structure, and the other was a ResNet structure combined with a pyramid network. Safdar et al. (76) used YOLO3 model to assess the effect of data augmentation methods. Carver et al. (82) adopted a 2D-UNET network to evaluate the segmentation performance of the synthetic image. Baid et al. (92) segmented glioma images with a 3D-UNET network. Prince et al. (29) adopted the long short-term memory (LSTM) network to realize the non-invasive diagnosis of adamantinomatous craniopharyngioma.

### 3.3. Hybrid Model

The quality of the data set is pivotal to the performance of AI algorithms. The input data can be the feature vectors extracted by the algorithm, or the raw data directly entered into the end-to-end learning system. The imaging features mentioned in section 3.1 can express and quantify the hidden information in the image, while their ability to describe the global information of images is insufficient, and the ability to filter noise is weak. By contrast, CNN itself as an excellent feature extractor, can obtain global high-order features (93). Therefore, some researches developed a hybrid model of traditional machine learning and deep learning, which could better match multi-source heterogeneous medical data and obtain more comprehensive information.

Deepak and Ameer (94) employed the modified GoogleNet to extract deep CNN features, and subsequently fed these features into SVM and KNN classifier models. The performance of classification for brain tumors was improved with the proposed hybrid model. Ning et al. (95) fused the radiomics features with depth features extracted by the CNN network, and performed feature selection to screen out the optimal feature subset. Finally, SVM was used for grading gliomas. The results demonstrated that integrating radiomics features and deep features for gliomas grading is feasible. Zhang et al. (96) combined the depth features extracted from the pre-trained CNN with texture features and morphological features, and evaluated the effects of these features by machine learning classifier. The experimental results suggested that the combination of features extracted by deep learning and radiomics is superior to a single modeling method. Li et al. (97) proposed a feature learning method based on generative adversarial network. AlexNet was used as feature extractor, while SVM was used for classification. The experimental results achieved high classification precision. Xia et al. (98) developed models based on deep learning and radiomics features respectively, and then applied an information-fusion method to fuse the prediction performance of the two models. The proposed fusion model improved the classification performance of non-invasive adenocarcinoma and invasive adenocarcinoma.

## 4. Discussion

### 4.1. Few-Shot Learning

At present, AI has achieved high performance in many fields, which rely on a mass of labeled samples and iterations of trained models. In the medical field, AI performs well in some scenarios where large amounts of training data are available, such as skin diseases and diabetic retinopathy (99, 100), etc. However, for most scenarios, the data collection and labeling are laborious and time-consuming. Besides that, some privacy ethics and obstacles are also difficult to overcome. All of these are real challenges for the applications of AI in the medical field.

According to the retrieved literature, the application of AI in craniopharyngioma has been emerging in the last 5 years. Data sets reported in most of the literature were from a single institution. The lack of standard databases and the small sample size of data sets affect the application of AI in diagnosis of craniopharyngioma. When experiments are carried out on small data sets, overfitting is inevitable and the generalization performance is often queried, especially in high-risk tasks like tumor diagnosis. Therefore, few-shot learning aiming to learn quickly from a small data set, is an issue worthy of further research. Several measures that can be implemented to deal with few-shot learning are discussed below.

(1) Data augmentation can expand a data set with transformation rules and prevent the over-fitting of the model. As a straightforward and simple solution, it has been commonly used in the retrieved literature. Common transformation rules include shift, rotation, scale, crop, flip, and other operations on a single sample. Other algorithms are also available to operate on multiple samples, such as SMOTE, mixup, etc. These methods with minor changes to the original images, can quickly and simply obtain a large amount of new data.

(2) Data synthesis can synthesize new data through neural networks, among which generated adversarial network (GAN) is the most representative one. The GAN framework includes generators and discriminators (101). The goal of the generator is to generate a large number of samples close to reality, while the discriminator should correctly distinguish between real samples and simulated samples. The game theory and confrontation training mode of GAN endow it with a strong ability in data augmentation.

(3) Feature enhancement enriches the diversity of samples by augmenting the sample feature space, and consequently expands the data sets. Typically, Schwartz et al. (102) developed a network comprised of encoders and decoders to generate new data. The encoder learned to extract the transferable deformations between pairs of samples of the same category in the data set, while the decoder learned how to apply these deformations to the samples in the new category to generate new data.

(4) Other more advanced strategies include meta-learning strategy, measurement learning strategy, parameter optimization strategy (103, 104). Meta-learning strategy is currently a novel research framework. In few-shot learning, meta-learning strategy learns meta-knowledge from prior tasks, and uses prior knowledge to guide the model to learn quickly. Through calculating the distance between the samples to be classified and the known ones, measurement learning strategy acquires the adjacent categories to determine the results of the samples to be classified. This algorithm does not need to fine-tune labeled images, but compares the image to be classified with the known ones to perform classification. Another problem of few-shot learning is that the generalization ability of the network deteriorates due to iterations of few parameters on small data sets, which results in the lack of credibility in the classification performance of the models. The parameter optimization strategy focuses on optimization of the basic learner through an optimizer, thus improving the credibility and generalization ability of the classifier.

(5) Transfer learning is a common technique used in deep learning frameworks. Considering that most of tasks are correlated, transfer learning can accelerate and optimize the learning efficiency of the model by transferring the pre-trained model parameters to the new model, rather than learning from scratch. For deep learning models, the transfer method applies the pre-training model to a new task by fine-tuning (105). For the transfer learning method, the source domain and the target domain do not need the same distribution of data, which overcomes the shortcomings of traditional machine learning, and has a great advantage in the case of few samples in the target domain and sufficient samples in related fields.

### 4.2. Classification of Imbalanced Data Sets

Most data sets in the medical field are unbalanced, that is, the sample size of one category is much smaller than that of another. The imbalanced data set may cause the neglection of the minority category samples leading to underfitting, or the overemphasis of the minority category samples leading to overfitting. In order to design the learning model with more reliable ability, it is necessary to address the problem of the imbalanced data set.

(1) Data pre-processing is an effective method to solve this problem. It can be performed by deleting the samples of the majority category or adding to the samples of the minority category to reduce the difference in the sample number. According to the difference in sampling methods, over-sampling, under-sampling, and a combination of the two methods are all feasible (106).

(2) In an imbalanced data set, the costs of misclassification on the majority and minority categories are different, and the misclassification of the minority category should cost higher. Based on this premise, cost sensitive methods are feasible methods to deal with an imbalanced data set, which assign different costs to misclassification of different categories by introducing cost matrix, and then construct classifiers with the goal of minimizing cost value (107).

(3) Additionally, the ensemble learning method is another measure to mitigate the influence of an imbalanced data set. Ensemble learning models combine several different weak learners together to form a strong learner. The generalization performance of the model is improved by taking advantage of the differences between each base learner. A typical example is Adaboost algorithm (108). In the training process, the algorithm will assign higher weight to the samples with a large prediction error, and the model will pay more attention to these samples in the next iteration.

### 4.3. Research on Semi-supervised Learning

In the medical field, manual labeled data are expensive and scarce, while a large number of unlabeled image data resources are left idle. The semi-supervised learning method based on a small number of labeled samples and a large number of unlabeled samples is more suitable in such real conditions. The recommended techniques for semi-supervised learning are as follows:

(1) Generative models as the early semi-supervised learning methods, establish the relationship between prediction models and unlabeled data based on clustering assumption and manifold assumption, and assume that all data are “generated” by the same potential model. Typical algorithms include Gaussian mixture model, Expectation-Maximum method, Naive Bayes method, and others (109–111).

(2) The self-training method is an incremental algorithm (112) that firstly uses a small number of labeled data to train an initial classification model, and then adopts this model to predict all unlabeled data. Only the samples with high confidence will be added to the training set for re-training, and the iterations are repeated until the termination condition is satisfied.

(3) The co-training method consists of several classification models. First, each base classifier is initialized with labeled data, and then the classifier selects the “most confident” unlabeled data, assigns the predicted category of samples and adds them to the labeled data set. The labeled data set is updated and provided to another classifier, and the iterations are repeated until each classifier does not change (113).

(4) The graph-based method maps the data set into a composition that presents the organizational relationship between the sample data. In the graph, the nodes of the graph represent the sample points, and the edges represent the similarity relationship between two sample points. And then the labels are spread from labeled data to unlabeled data based on the adjacency relationship on the graph (114).

(5) Semi-supervised SVM is an extension of SVM on semi-supervised learning (115). After adding unlabeled data into the feature space, semi-supervised SVM tries to find a partitioning hyperplane that still divides the labeled data and passes through the low-density region of the samples.

### 4.4. Multi-Omics Model Research

Traditional single omics has some limitations in interpreting disease due to its complexity. The development of multi-omics technology provides abundant materials for the study of complex diseases. Genomics, epigenomics, transcriptomics, proteomics, and metabolomics are all important components of systems biology. Multi-omics research can study diseases more comprehensively by integrating the data of different omics effectively. Adding some clinical parameters such as gender and age to the feature set is a preliminary attempt. In addition, Guo et al. (116) graded glioma by utilizing radiomics and clinical parameters, such as age and markers of inflammation in the blood. Chen et al. (117) combined histology images and genomics to predict survival outcomes, and the results performed higher than single omics experiments. In the future, it will be an important research direction that comprehensively analyzes complex diseases like cancers by combining multi-omics data.

## 5. Conclusion

With the successful applications of AI in many fields, research on the application of AI in diagnosis of craniopharyngioma has emerged in recent years. This study reviewed the existing applications of artificial intelligence in craniopharyngioma from the aspects of differential classifications, brain tissue invasiveness and gene mutation prediction, and postoperative prediction. Leveraging the relevant literature on other similar tumors, the artificial intelligence-based diagnostic routes were further proposed. Traditional machine learning methods are more explanatory and less computation is required. Intelligent diagnosis of craniopharyngioma based on traditional machine learning included steps of image preprocessing, image segmentation, feature extraction, feature selection, machine learning modeling. Deep learning model is an end-to-end learning framework, which heavily relies on a mass of data. Therefore, diagnosis of craniopharyngioma based on deep learning is usually included steps of data augmentation and design of model. The study also proposed the methods that could be adopted in each step. The applications of artificial intelligence technology in the diagnosis of craniopharyngioma are still in the preliminary period. The lack of standard data sets and small data sets may affect the development of artificial intelligence technology in this field. In view of the existing research, this study discussed the attentions required for future research. Few-shot learning is one of the first works to be addressed. Data augmentation, data synthesis, feature enhancement, some advanced learning strategies, and transfer learning are available measures for learning on a small data set. In addition, future research should also pay attention to the problem of imbalanced data sets. Over-sampling or undersampling technology, cost sensitive method, and ensemble learning methods are recommended solutions. Another research direction should point to the semi-supervised learning model which is a suitable choice for the medical field with scarce labeled data. Additionally, multi-omics fusion mode can better describe complex diseases like cancers.

## Author Contributions

CQ wrote the manuscript draft, conducted the review of literature, and summarized the findings of the review. XW, WH, and XM discussed the review and edited the manuscript. All authors contributed to the article and approved the submitted version.

## Funding

This work has been partially supported by the National Key Research programs of China (2016YFA0100900, 2016YFA0100902), the Chinese National Natural Science Foundation Projects #82090051 #81871442, the Youth Innovation Promotion Association CAS(#Y201930), the Fujian Province Natural Science Foundation (2017J01779), and in part by the Shandong Province Natural Science Foundation (ZR2020KF016). Additionally, this work is partially supported by Fujian Key Lab of Agriculture IOT Application, IOT Application Engineering Research Center of Fujian Province Colleges and Universities, Digital Fujian Research Institute for Industrial Energy Big Data.

## Conflict of Interest

The authors declare that the research was conducted in the absence of any commercial or financial relationships that could be construed as a potential conflict of interest.

## Publisher's Note

All claims expressed in this article are solely those of the authors and do not necessarily represent those of their affiliated organizations, or those of the publisher, the editors and the reviewers. Any product that may be evaluated in this article, or claim that may be made by its manufacturer, is not guaranteed or endorsed by the publisher.
